# Assessing Microstructural and Molecular Brain Changes in Early Cognitive Decline Using Multiparametric 3T Magnetic Resonance Imaging

**DOI:** 10.3390/neurolint18090177

**Published:** 2026-09-18

**Authors:** Zongpai Zhang, Jingpu Wu, Isabel M. Rios Pulgar, Elizabeth H. T. Chang, Keyi Chai, Puyang Wang, Shanshan Jiang, Xu Li, Kenichi Oishi, Gwenn S. Smith, Carrie Wagandt, Gregory M. Pontone, Abhay Moghekar, Arnold Bakker, Jinyuan Zhou

**Affiliations:** 1 Department of Radiology, School of Medicine, Johns Hopkins University, Baltimore, MD 21287, USA; 2 Department of Electrical and Computer Engineering, Whiting School of Engineering, Johns Hopkins University, Baltimore, MD 21211, USA; 3 Department of Psychiatry and Behavioral Sciences, School of Medicine, Johns Hopkins University, Baltimore, MD 21287, USA; 4 F. M. Kirby Research Center for Functional Brain Imaging, Kennedy Krieger Institute, Baltimore, MD 21205, USA; 5 Department of Neurology, School of Medicine, Johns Hopkins University, Baltimore, MD 21224, USA

**Keywords:** multiparametric MRI, CEST imaging, APT imaging, mild cognitive impairment, Alzheimer’s disease, microstructural alteration

## Abstract

**Objectives**: Developing sensitive imaging biomarkers is critical for capturing early brain alterations in Alzheimer’s disease. This exploratory study aimed to evaluate how multiparametric magnetic resonance imaging (MRI) measures, including chemical exchange saturation transfer (CEST) imaging, can detect early cognitive impairment and to compare their diagnostic performance across multiple brain regions. **Methods**: 18 cognitively normal participants and 18 patients with mild cognitive impairment or mild dementia were recruited to undergo CEST and other MRI sequences. Imaging metrics included CEST measures (amide proton transfer-weighted or APTw, APT^#^, nuclear Overhauser enhancement or NOE^#^), alongside conventional measures (T_1_, T_2_) and advanced measures (magnetization transfer ratio or MTR, quantitative susceptibility mapping or QSM, apparent diffusion coefficient or ADC). Quantitative analyses were performed in the hippocampus, caudate, putamen, posterior cingulate cortex, and amygdala. Associations between MRI measures and cognition were evaluated using Pearson correlation analysis, and the diagnostic performance of single-parameter and multiparameter models were assessed using logistic regression and receiver operating characteristic analysis. **Results**: Among all evaluated MRI measures, APTw, APT^#^, NOE^#^, MTR, T_1_, and QSM demonstrated statistically significant group differences (cognitively normal vs. mild cognitive impairment or mild dementia), with medium to very large effect sizes in at least one region. Across all evaluated regions, APT^#^ and NOE^#^ signals were consistently and positively associated with clinical dementia severity (based on the Clinical Dementia Rating), whereas MTR showed consistently negative associations. In contrast, APTw, T_1_, T_2_, QSM, and ADC exhibited weaker or more region-dependent associations. Correlations with the Mini-Mental State Examination were generally weaker across MRI measures and regions. Among these, APT^#^ showed the strongest and most consistent associations with clinical severity, and was the best single-parameter classifier in most regions. **Conclusions**: Multiparametric MRI with CEST imaging shows potential for detecting microstructural and molecular alterations associated with cognitive dysfunction in individuals at risk for early cognitive decline.

## 1. Introduction

Alzheimer’s disease (AD) is a progressive neurodegenerative disorder and a leading cause of cognitive decline in older adults. Early identification of cognitive and associated brain changes, particularly at the stage of mild cognitive impairment (MCI), is critical for improving disease characterization and enabling timely intervention [1,2]. Importantly, MCI is a clinically and etiologically heterogeneous syndrome and is not specific to AD. Although AD represents a common underlying cause, MCI may also precede or accompany other neurodegenerative conditions, including Lewy body disease, frontotemporal dementia, and vascular cognitive impairment [3,4]. In AD, cognitive decline is associated with a complex cascade of pathological processes, including the accumulation of amyloid-β and tau, synaptic and neuronal dysfunction, neuroinflammation, vascular alterations, and progressive neurodegeneration [5,6]. These processes evolve across spatially and temporally heterogeneous brain regions, providing a pathophysiological rationale for imaging approaches that probe complementary molecular, microstructural, and structural tissue properties. In addition, systemic and metabolic factors may contribute to regional brain vulnerability and imaging alterations in cognitive disorders. For example, hypoglycemic episodes and glycemic variability have recently been recognized as potential contributors to cerebral hypoperfusion, including hippocampal involvement, in selected neurodegenerative conditions [7,8,9].

Clinical assessment tools such as the Clinical Dementia Rating (CDR) and the Mini-Mental State Examination (MMSE) are widely used to quantify cognitive impairment and disease severity [10]. However, these measures provide limited insight into the mechanisms of regional brain pathology. Molecular imaging techniques such as amyloid-β (Aβ) and tau positron emission tomography (PET) provide in vivo assessment of the core pathological features of AD. Amyloid PET enables visualization of fibrillar Aβ plaque burden and is an established biomarker for determining the presence of AD-related amyloid pathology, whereas tau PET provides complementary information on the regional distribution of aggregated tau pathology and is particularly informative for biological staging and assessment of disease severity. Nevertheless, PET imaging remains limited by factors including cost, radiation exposure, and restricted accessibility. These limitations motivate the development of complementary, non-ionizing MRI-based approaches that can probe tissue alterations associated with early cognitive decline [5,11].

Conventional structural MRI has played an important role in the assessment of AD, primarily by measuring brain atrophy and regional volume loss [12,13]. Notably, advanced quantitative MRI sequences can detect early neurodegenerative changes and other downstream biological consequences, including microstructural damage and metabolic alterations. In addition to T_1_ and T_2_ relaxation times, advanced MRI metrics such as the semi-solid magnetization transfer ratio (MTR) [14], quantitative susceptibility mapping (QSM) [15], and apparent diffusion coefficient (ADC) [16] can provide insights into macromolecular integrity, iron deposition, and microstructural organization. These measures have been increasingly applied in AD research and have demonstrated regional alterations associated with AD pathology. Multiparametric MRI has been widely used to assess diverse tissue properties by integrating complementary contrasts [17,18]. Nevertheless, these contrasts remain indirect markers of AD pathology, and their sensitivity and specificity may vary across brain regions, reflecting the heterogeneous nature of AD-related changes [19,20]. Because these morphological and structural MRI metrics often lack the sensitivity to capture the early AD pathological processes [21], there is a critical need for advanced MRI approaches capable of detecting molecular-level brain changes associated with cognitive decline.

Chemical exchange saturation transfer (CEST) MRI [22,23], including amide proton transfer (APT) and nuclear Overhauser enhancement (NOE), is an advanced molecular imaging technique. It detects low-concentration biomolecules and characterizes microenvironmental tissue properties by measuring water signal attenuation driven by proton exchange with saturated target protons (like amide, amine, or hydroxyl groups). A number of biomedical applications have been explored successively, including various cancers [24,25,26,27] and neurological disorders [28,29,30,31,32]. Notably, APT imaging has been proposed as a sensitive quantitative molecular MRI biomarker for detecting soluble Aβ and tau oligomers characteristic of AD [33,34,35], and elevated APT or APT-weighted (APTw) signals have been reported in previous human AD studies [36,37,38,39]. Current amyloid PET tracers are primarily designed to detect Aβ plaques and tau tangles, rather than more toxic, APT-sensitive soluble oligomers. Despite their potential relevance to AD, systematic investigations of CEST measures across multiple brain regions, their relationships with cognitive impairment, and their comparative performance relative to other quantitative MRI-derived measures remain limited.

This study aimed to systematically evaluate the associations between multiparametric MRI biomarkers and cognitive decline across multiple brain regions. We focused on three CEST MRI metrics (APTw, APT^#^ and NOE^#^) obtained from a so-called robust two-step extrapolated semi-solid magnetization transfer reference fitting (rEMR) method [39] and compared their performance in discriminating between cognitively normal (CN) and MCI or mild dementia participants with conventional structural and other advanced MRI measures. We hypothesized that molecular APT^#^ and NOE^#^ would demonstrate stronger associations with cognitive impairment and superior classification performance, reflecting their sensitivity to early brain changes before obvious structural atrophy.

## 2. Materials and Methods

### 2.1. Patient Recruitment

This study was approved by the local Institutional Review Board, and informed, written consent was obtained from each subject. MCI was defined by core clinical criteria for the diagnosis of MCI following Albert et al. [40]. AD dementia was defined by core clinical criteria for probable AD dementia as outlined by McKhann et al. [41]. Subjects were between 50 and 90 years old. Four mild dementia patients (66.0 ± 10.0 years old), fourteen MCI patients (69.7 ± 8.6 years old), and eighteen age-matched, CN healthy volunteers (70.3 ± 7.8 years old) were recruited for this study according to the inclusion criteria. Due to the limited amount of data, eighteen mild dementia and MCI patients were combined as the MCI/mild dementia group (68.9 ± 8.8 years old) and compared to the healthy volunteers (the CN group). Medical history, including cardiovascular, cerebrovascular, neurological, metabolic, endocrine, and other relevant medical comorbidities, was systematically collected from all participants as part of the clinical assessment. Participants were excluded if they had a history of major neurological disease, including major stroke, brain or spinal cord tumor, or traumatic brain injury; a history of major psychiatric disorders, such as schizophrenia or bipolar disorder; or contraindications to MRI, including claustrophobia, body size incompatible with the scanner, cardiac pacemakers or neurostimulators, intracranial clips or other incompatible metallic implants, or metallic foreign bodies in the eyes. For participants with MCI or AD dementia, additional exclusion criteria included the presence of REM sleep behavior disorder, a Movement Disorder Society-Unified Parkinson’s Disease Rating Scale Part III (MDS-UPDRS-III) score ≥10, or visual hallucinations. Pregnant women were also excluded.

### 2.2. MRI Data Acquisition

MR imaging was performed on a 3T MRI scanner (Achieva dStream, R5.7.1.2; Philips Healthcare, Best, The Netherlands), using a body coil for excitation and a 32-channel phased-array head coil for signal reception. APT imaging was performed using a time-interleaved, parallel transmission-based radiofrequency saturation module that consisted of 30 single-lobe sinc-Gaussian saturation pulses (50 ms each, duty cycle = 100%). 3D dynamic images with 24 frequency offsets (80, 60, 40, 30, 20, 10, 8, ±4, ±3.5, ±3.5, ±3, ±2.5, ±2, ±2, ±1.5 ppm, and M_0_) were acquired. Imaging parameters were as follows: repetition time (TR) = 5 s, echo time (TE) = 5.4 ms, saturation power = 1.5 μT, saturation duration = 1.5 s, 15 slices, slice thickness = 4 mm, field of view (FOV) = 212 × 192 mm^2^, acquisition matrix = 120 × 107, reconstructed to 0.83 × 0.83 mm^2^ in-plane resolution, and acquisition time = 4 m 10 s. The 60-mm APT sequence consistently covered the entire hippocampus. B_0_ inhomogeneity correction was performed using B_0_ maps acquired with a water saturation shift referencing sequence [42] (TR = 1152 ms, TE = 5.4 ms, saturation power = 0.5 μT, saturation duration = 0.4 s, 15 slices, slice thickness = 4 mm, acquisition time = 40 s).

The MRI protocol also included the following sequences: T_1_-weighted (T_1_w) using Magnetization Prepared Rapid Gradient Echo (TR = 6.3 ms, TE = 2.9 ms, inversion time [TI] = 786 ms, isotropic voxel size = 1.0 × 1.0 × 1.0 mm^3^); 3D Fluid-Attenuated Inversion Recovery (FLAIR) (TR = 4.8 s, TE = 232 ms, TI = 1.65 s, isotropic voxel size = 1.0 × 1.0 × 1.0 mm^3^, 192 slices, thickness = 1 mm); T_1_ mapping using a 3D Look-Locker sequence with 38 inversion times (TI = 42.5 ms, ΔTI = 157.6 ms, TR = 3.1 ms, TE = 1.58 ms, flip angle = 4°, 15 slices, slice thickness = 4 mm); T_2_ mapping using a multi-echo spin-echo sequence (TR = 3000 ms, TE = 30, 60, 90, 120, and 150 ms, 15 slices, slice thickness = 4 mm); susceptibility-weighted imaging (SWI) using a multi-echo gradient-echo sequence (TR = 40 ms, 1st TE/ΔTE = 6/6 ms, 5 echoes, 132 slices, isotropic voxel size = 1.0 × 1.0 × 1.0 mm^3^); and diffusion-weighted imaging (DWI) (TR = 3913 ms, TE = 79 ms, b value = 1000 s/mm^2^, 33 slices, slice thickness = 3.4 mm, inter-slice gap = 0.6 mm).

### 2.3. Data Processing

T_1_, T_2_, and ADC maps were generated from 3D Look-Locker, multi-echo spin-echo, and DWI data according to established methods or automatically using the scanner software [16,43]. QSM maps were derived from multi-echo gradient-echo data. QSM was initially reconstructed in its native space (1-mm isotropic resolution) using the Structural Feature-based Collaborative Reconstruction algorithm with automatic referencing to cerebrospinal fluid (SFCR + 0), as implemented in the QSM Toolbox (v3.0), following previously established protocols [44,45,46]. To perform multiparametric analysis alongside the lower-resolution CEST sequence (4-mm slice thickness, oblique orientation), spatial alignment was achieved using the reg_aladin tool from the NiftyReg package. Specifically, the MRI maps underwent 3D rigid-body co-registration and were forward-resampled onto the APT image grid via trilinear interpolation. This automated registration pipeline directly accounts for the slice thickness mismatch and volume-averaging effects between modalities, ensuring precise voxel-by-voxel alignment without manual projection bias. Total intracranial volume (TIV) was calculated using T_1_w images.

All CEST dynamic images (including those from the water saturation shift referencing sequence) were co-registered to the M_0_ images using FreeSurfer R7.4.1 and NiftyReg R1.5.76. Following B_0_ correction, APTw images at 3.5 ppm [47] and MTR images at 20 ppm were calculated using standard approaches. A recently developed, robust two-step extrapolated semi-solid magnetization transfer reference fitting method was used for the quantification of APT^#^ and NOE^#^ [39]. In the first step, the B_0_-corrected Z-spectrum (Z_exp_) was fitted across all frequency offsets (including small offsets near 0 ppm), alongside T_1_ and T_2_ maps as inputs, to estimate parameters of the direct saturation and magnetization transfer contrast pools ($T_{1}^{a}$, $T_{2}^{a}$, $T_{2}^{b}$, R, $M_{0}^{b}$, and a bias term). In the second step, $T_{1}^{a}$, $T_{2}^{a}$ and the bias term were fixed, while $T_{2}^{b}$, R, and $M_{0}^{b}$ were fine-tuned using only the larger positive offsets. Using the fitted parameters, the extrapolated reference Z-spectrum (Z_EMR_) was calculated to account for direct saturation and magnetization transfer contrast effects. Finally, residual signals at ±3.5 ppm were defined as APT^#^ = Z_EMR_(+3.5 ppm) − Z_exp_(+3.5 ppm) and NOE^#^ = Z_EMR_(−3.5 ppm) − Z_exp_(−3.5 ppm). Quantitative APT^#^ has been shown to improve detection specificity and diagnosis performance by reducing confounding effects [39].

**Figure 1:**
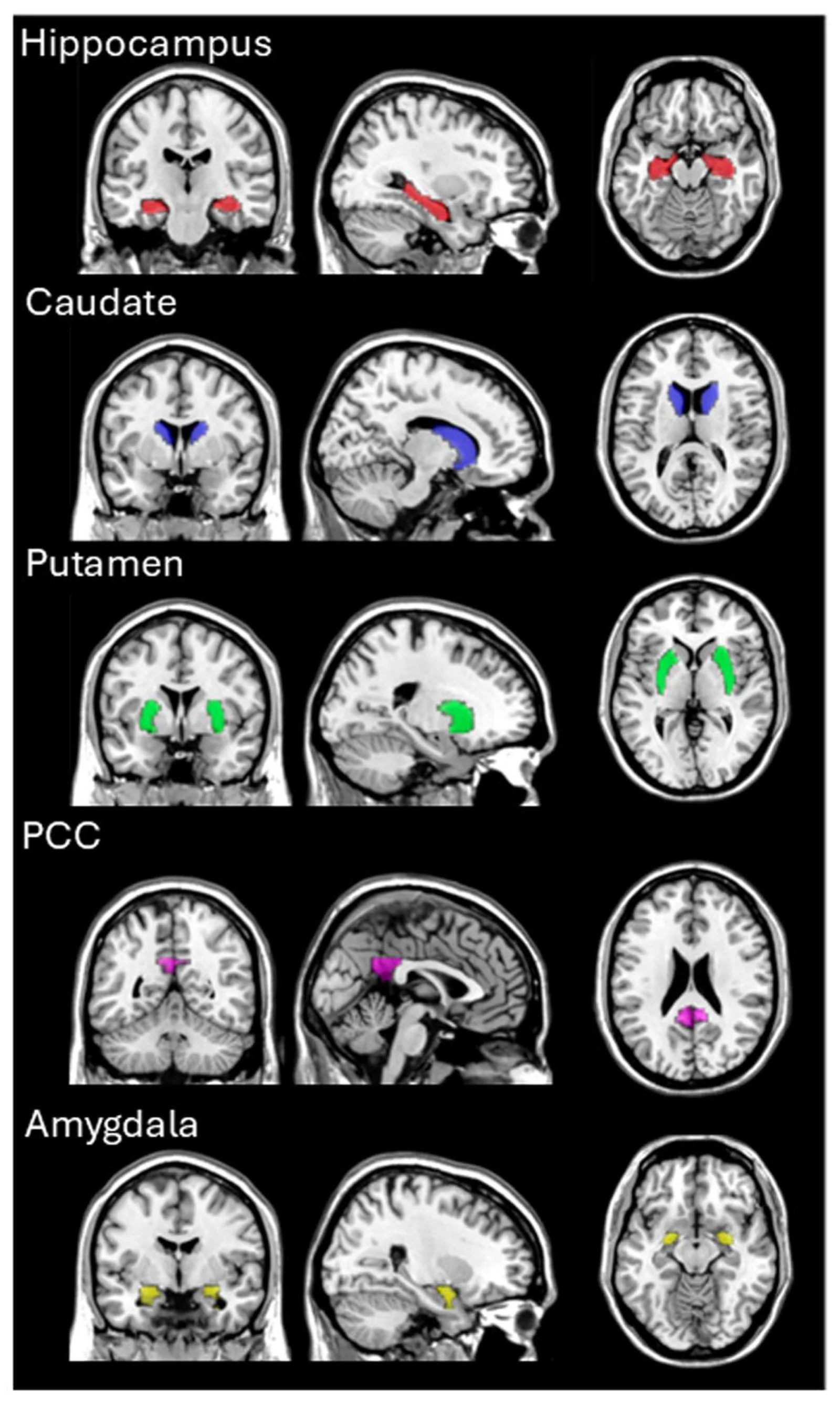
Representative T_1_w image showing the locations of the five ROIs: hippocampus, caudate, putamen, PCC, and amygdala.

After all MRI parameters were co-registered and corrected, manual skull stripping and region of interest (ROI) segmentation were performed using the EMR M_0_ and T_1_ weighted images as references. ROIs encompassing the hippocampus, caudate, putamen, posterior cingulate cortex (PCC), and amygdala (Figure 1) were manually drawn by one researcher (Z.Z.) and confirmed by a second researcher (K.O.) using ITK-SNAP [48]. This ensured that each ROI could be consistently applied across all parameters, which shared the same spatial geometry (15 slices, 4 mm slice thickness, matched in plane resolution). These ROIs were selected a priori to sample complementary medial temporal, cortical, and subcortical regions implicated in cognitive function and neurodegenerative changes. The hippocampus and amygdala were included because of their established roles in memory and their vulnerability in early cognitive decline, while the PCC was selected because of its involvement in memory-related and default-mode network function. The caudate and putamen were included to assess subcortical alterations and to extend the evaluation beyond medial temporal and cortical regions [49,50,51].

### 2.4. Cognitive Tests

The CDR, including the Global Score (CDR-GS) and Sum of Boxes (CDR-SB), and the MMSE were used to assess cognitive function and dementia severity of all participants [52]. The CDR is a clinician-administered scale that evaluates dementia severity across six cognitive and functional domains [53]. A CDR-GS of 0, 0.5, 1, 2, or 3 corresponds to no, very mild, mild, moderate, or severe dementia, respectively. The CDR-SB ranges from 0–18 with 0 (normal), 0.5–4.0 (MCI), 4.5–9.0 (mild dementia), 9.5–15.5 (moderate dementia), and 16.0–18.0 (severe dementia). The MMSE is a brief cognitive screening tool consisting of 11 items that assess five cognitive domains, with a maximum score of 30. Scores of 24 or higher indicate normal cognitive function, whereas scores of 19–23, 10–18, and 9 or below correspond to mild, moderate, and severe dementia, respectively [54,55]. Psychiatric history, including self-reported history of major depressive disorder and anxiety disorder, was collected as part of the clinical assessment. Depressive symptoms were additionally assessed using the 15-item Geriatric Depression Scale (GDS-15). Scores of 0–4 were considered within the non-depressed range, whereas scores of 5–8 were considered indicative of mild depressive symptoms [56].

### 2.5. Statistical Analysis

Continuous variables were summarized as mean ± standard deviation for approximately normally distributed data and as median [interquartile range (IQR)] for non-normally distributed data, as appropriate. Differences between groups in GDS-15 scores were assessed using the Mann–Whitney U test. Group differences within each ROI were assessed using independent-samples *t*-tests and Cohen’s *d* effect sizes, defined as small (0.2 ≤ |d| < 0.5), medium (0.5 ≤ |d| < 0.8), large (0.8 ≤ |d| < 1.3), or very large (|d| ≥ 1.3). Associations between ROI-based MRI measures and cognitive scores, including CDR and MMSE, were evaluated with Pearson correlation analysis, defined as weak (0.1 ≤ |r| < 0.3), moderate (0.3 ≤ |r| < 0.5), and strong (|r| ≥ 0.5) correlations. TIV was compared between groups to confirm the absence of systematic differences in brain size. Multiple comparisons were controlled using the false discovery rate (FDR), with a corrected *p* < 0.05 considered statistically significant. To assess whether depressive symptoms influenced the cognitive findings, additional sensitivity analyses were performed. The difference between groups in MMSE was evaluated using linear regression with group and GDS-15 score as predictors. Associations between images and cognition were additionally reassessed using partial Pearson correlations controlling for GDS-15 score among participants with available GDS-15 data. Missing GDS-15 values were handled using complete-case analysis. FDR correction was applied to the partial correlation analyses to account for multiple comparisons.

The discriminative performance of each individual parameter and multiparametric combinations was evaluated using logistic regression. All imaging features were standardized using z-score normalization prior to model fitting. Single-parameter models were assessed using the receiver operating characteristic (ROC) analysis. For each single-parameter model, 95% confidence intervals were estimated using nonparametric bootstrap resampling (2000 iterations), and 10-fold cross-validation was performed to calculate the mean and standard deviation of the area under the ROC curve (AUC). For each ROI, the single MRI feature yielding the highest AUC among all single-parameter models was defined as the BestSingle predictor. Further, full multiparametric logistic regression models incorporating all available MRI features within each ROI were constructed to assess combined discriminative performance. Finally, feature selection was performed using Least Absolute Shrinkage and Selection Operator (LASSO)-regularized logistic regression with cross validation to identify a sparse subset of informative predictors. When one or more features were selected by LASSO, a logistic regression model based on the selected features was fitted and evaluated. In some ROIs, LASSO did not select any predictors under cross validation, indicating that no individual feature provided a stable improvement in model fit beyond the intercept only model.

Statistical comparisons of AUCs between models were performed using the DeLong test, including comparisons between the full multiparametric model and the BestSingle model, as well as between the LASSO-selected model and the BestSingle model. When class sample sizes were small or DeLong test assumptions were violated, a bootstrap-based resampling approach was used as a fallback to estimate the statistical significance of AUC differences. LASSO was used for feature selection in a predictive modeling framework and does not provide statistical inference. Therefore, multiple comparison correction was applied only to statistical tests involving group comparisons, correlation analyses and AUC comparisons. All analyses were conducted separately for each ROI to preserve regional interpretability and mitigate overfitting in the context of limited sample size, thereby enabling ROI-specific evaluation of predictive accuracy and statistical significance. Given the modest sample size, the present analyses were considered exploratory and hypothesis-generating. Accordingly, emphasis was placed on effect sizes, confidence intervals, consistency of findings across regions and related MRI measures, and cross-validated classification performance, rather than on statistical significance alone. Analyses were implemented in MATLAB R2024b and Python R3.12.4 with NumPy and SciPy.

## 3. Results

### 3.1. Participant Characteristics and Clinical Profiles

A total of 36 participants were included in this study, comprising 18 healthy volunteers in the CN group and 18 patients in the MCI/mild dementia group (Table 1). The two groups were well-matched for age and gender. The mean age was 70.3 ± 7.8 years for the CN group and 68.9 ± 8.8 years for the MCI/mild dementia group (*p* = 0.606). The gender distribution was also similar between groups, with 5 males and 13 females in the CN group, and 7 males and 11 females in the MCI/mild dementia group (*p* = 0.479). Furthermore, no significant difference was found in TIV, with the CN group having a mean of 1363.1 ± 130.9 mL and the MCI/mild dementia group having a mean of 1406.5 ± 142.3 mL (*p* = 0.33).

Significant differences were observed in clinical and cognitive measures. All participants in the MCI/mild dementia group had a CDR-GS ≥ 0.5, whereas all CN participants had a CDR-GS score of 0 (*p* < 0.001). Similarly, CDR-SB scores were significantly higher in the MCI/mild dementia group than in the CN group (*p* < 0.001). The median CDR-SB score in the MCI/mild dementia group was 1.75 (range, 0.5–7.0), indicating overall mild clinical impairment as compared with 0 (range, 0–0.5) in the CN group. A significant difference in MMSE scores was also observed between the two groups. The mean MMSE score for the CN group was 28.8 ± 1.4, while the MCI/mild dementia group scored significantly lower with a mean of 25.9 ± 3.5 (*p* = 0.004). These results confirm that while the two groups were comparable in basic demographic and morphological characteristics, the MCI/mild dementia group exhibited substantial cognitive impairment consistent with clinical diagnosis. Medical comorbidities were also assessed in both groups, including hypertension, hypercholesterolemia, vitamin B12 deficiency, and thyroid disease, with no documented diagnosis of diabetes in either group. No significant between-group differences were observed in the reported comorbidities. GDS-15 scores were higher in the MCI/mild dementia group than in the CN group (median [IQR], 2 [1–3] vs. 0.5 [0–2], *p* = 0.032). However, 16 of 18 participants (88.9%) in the MCI/mild dementia group and all 18 CN participants had GDS-15 scores within the 0–4 range; only two participants in the MCI/mild dementia group had scores within the mild depressive symptom range (5–8). The prevalence of self-reported history of major depressive disorder and anxiety disorder did not differ significantly between groups (Table 1).

### 3.2. Group Differences in MRI Measures Between CN and MCI/Mild Dementia

**Figure 2:**
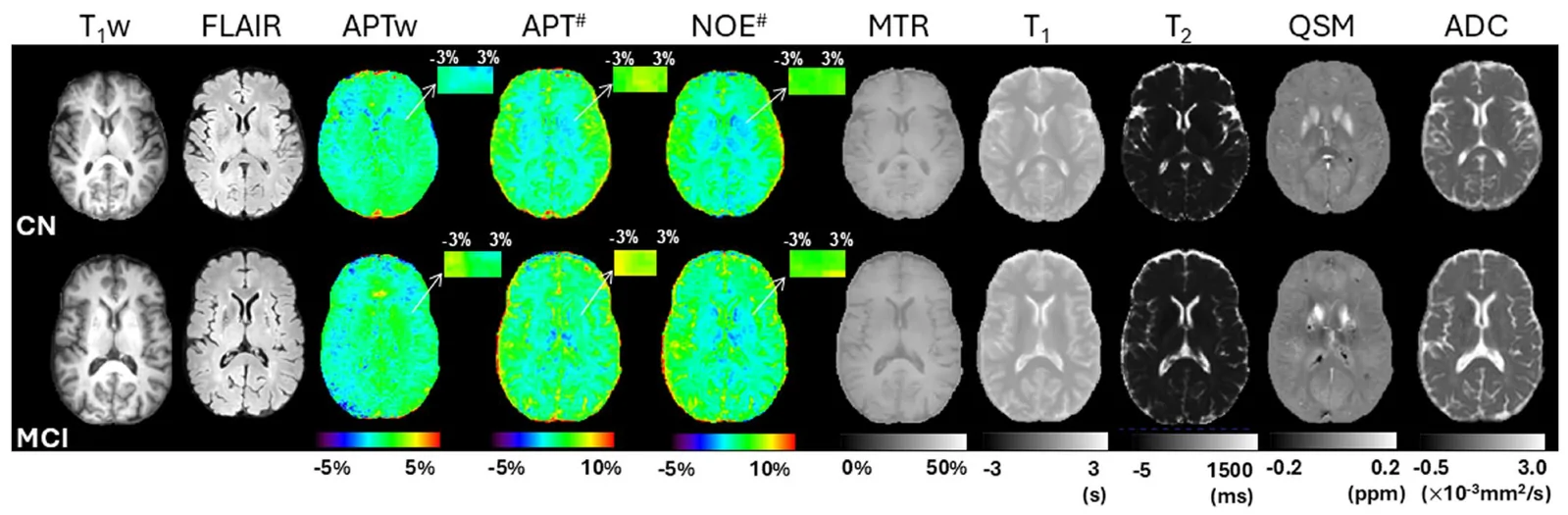
Two examples of multiparametric MR images of CN and MCI/mild dementia participants. CN: 68 years old, Female, MMSE = 27; MCI: 61 years old, Female, MMSE = 19. Higher APTw, APT^#,^ and NOE^#^ signals can be seen in MCI, particularly with small windows (insets).

**Figure 3:**
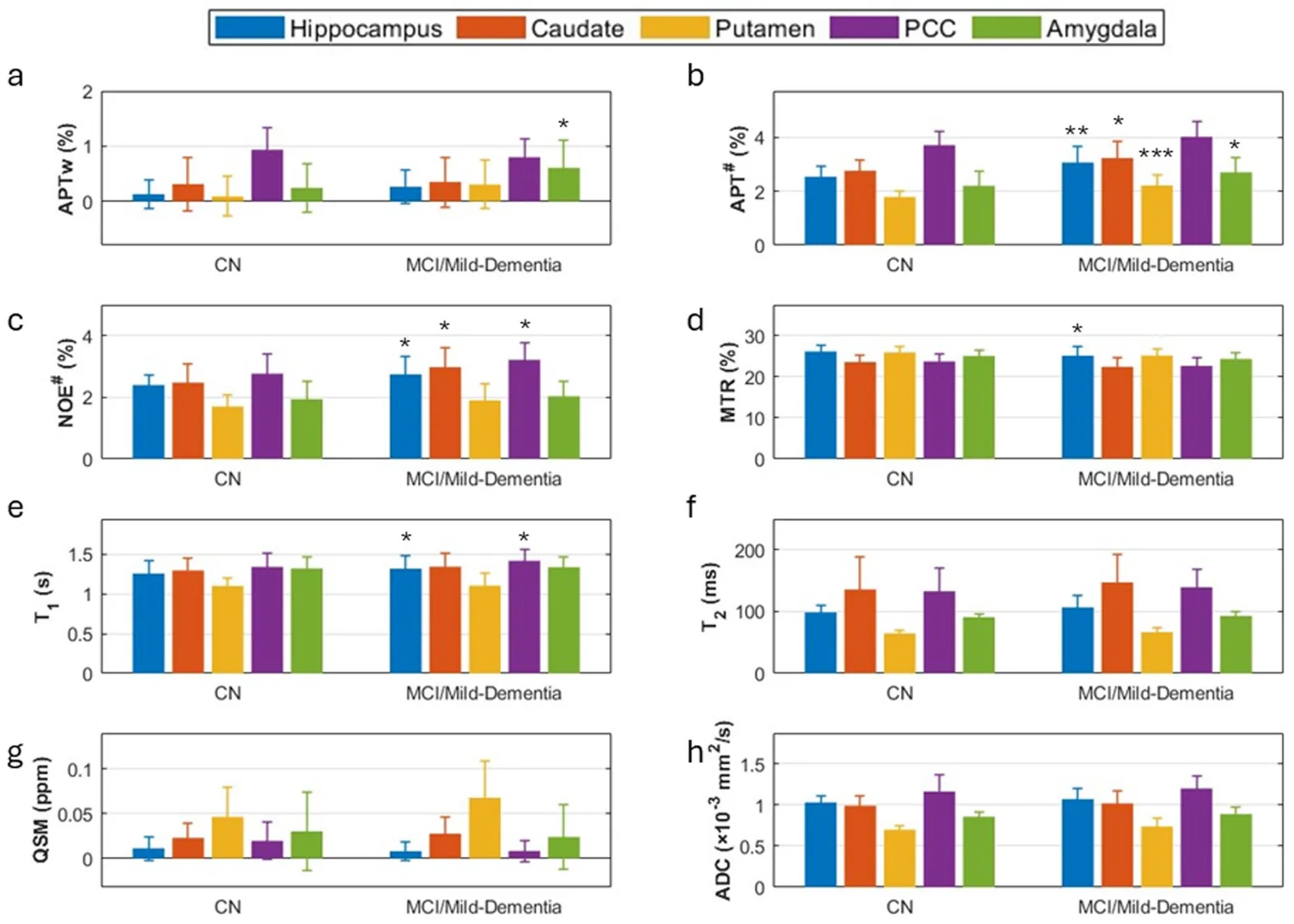
Comparison of MRI measures across five ROIs between CN and MCI/mild dementia groups. (**a**) APTw; (**b**) APT^#^; (**c**) NOE^#^; (**d**) MTR; (**e**) T_1_; (**f**) T_2_; (**g**) QSM; and (**h**) ADC. Asterisks above bars indicate statistically significant differences between CN and MCI/mild dementia groups for the corresponding ROI (* *p* < 0.05, ** *p* < 0.01, *** *p* < 0.001).

Figure 2 provides two representative examples of multiparametric MR images for a CN and an MCI subject, demonstrating slight visual alterations on CEST images between the two groups. Figure 3 illustrates the group differences across five evaluated ROIs, with asterisks indicating statistically significant contrasts between the groups. Notably, three CEST measures (APTw, APT^#^, and NOE^#^) demonstrated consistent alterations across multiple ROIs, highlighting the APT^#^ metric as the most sensitive measure for distinguishing CN from MCI/mild dementia participants.

Cohen’s d effect sizes for comparisons between CN and MCI/mild dementia participants across five ROIs are summarized in Table 2. Among all MRI measures, APT^#^ demonstrated the largest and most consistent group differences, showing large to very large effect sizes in the hippocampus (d = 1.08, *p* = 0.007), caudate (d = 0.95, *p* = 0.013), putamen (d = 1.52, *p* < 0.001), and amygdala (d = 0.94, *p* = 0.011). NOE^#^ also showed significant group differences in the hippocampus (d = 0.80, *p* = 0.047), caudate (d = 0.79, *p* = 0.031), and PCC (d = 0.76, *p* = 0.037), although its effect sizes were medium to large or non-significant in the putamen and amygdala. APTw demonstrated a significant difference only in the amygdala (d = 0.75, *p* = 0.031). MTR also had a significant difference in the hippocampus (d = −0.75, *p* = 0.049) while T_1_ showed similar results in both hippocampus (d = 0.82, *p* = 0.022) and PCC (d = 0.79, *p* = 0.026). In contrast, conventional and other advanced MRI measures, namely, T_2_ and ADC, generally exhibited small to medium effect sizes and did not reach statistical significance across all ROIs. QSM showed a significant difference in PCC (d = −0.73, *p* = 0.039) and moderate effect sizes in the putamen (d = 0.62, *p* = 0.072), with a trend towards significance, suggesting region-dependent susceptibility alterations.

### 3.3. Associations Between Cognitive Scores and ROI-Based MRI Measures

Correlations between MRI measures in five ROIs and cognitive scores (CDR-GS, CDR-SB, and MMSE) are summarized in Table 3. In the hippocampus, both APT^#^ and NOE^#^ were significantly and positively correlated with CDR-GS (r = 0.55, *p* = 0.008; r = 0.48, *p* = 0.021, respectively) and CDR-SB (r = 0.58, *p* = 0.003; r = 0.53, *p* = 0.006). Additionally, T_1_ showed a significant positive correlation with CDR-GS (r = 0.41, *p* = 0.034), and T_2_ showed significant positive correlations with both CDR-GS and CDR-SB (r = 0.38, *p* = 0.036; r = 0.40, *p* = 0.032). Conversely, MTR was significantly and negatively correlated with CDR scores (r = −0.39, *p* = 0.036; r = −0.41, *p* = 0.032). Additionally, QSM was significantly and positively associated with MMSE (r = 0.38, *p* = 0.047). Other measures showed weaker or non-significant associations.

In the caudate, both APT^#^ and NOE^#^ were positively correlated with CDR-GS (r = 0.42, *p* = 0.021; r = 0.43, *p* = 0.019, respectively) and CDR-SB (r = 0.38, *p* = 0.045; r = 0.37, *p* = 0.047), whereas MTR showed a negative correlation with CDR-GS (r = −0.38, *p* = 0.049). In the putamen, APT^#^ and QSM exhibited significant correlations with CDR-GS (r = 0.57, *p* = 0.004; r = 0.39, *p* = 0.048) and CDR-SB (r = 0.50, *p* = 0.022; r = 0.39, *p* = 0.046). In addition, T_2_ was positively correlated with MMSE (r = 0.39, *p* = 0.044), while QSM was negatively correlated with MMSE (r = −0.55, *p* = 0.001). For the PCC, NOE^#^ was positively correlated with CDR-GS (r = 0.38, *p* = 0.041). Interestingly, APTw showed a significant negative correlation with CDR-SB (r = −0.35, *p* = 0.0498 ~ 0.050), indicating lower APTw values in individuals with greater clinical severity. Finally, in the amygdala, APTw was positively associated with CDR-GS (r = 0.38, *p* = 0.043) and APT^#^ was positively associated with CDR-GS (r = 0.47, *p* = 0.044) and CDR-SB (r = 0.45, *p* = 0.047). MTR was negatively associated with CDR-GS (r = −0.40, *p* = 0.043) and CDR-SB (r = −0.39, *p* = 0.043).

In sensitivity analyses, the differences between groups in MMSE remained significant after adjustment for GDS-15 score (adjusted group coefficient = −2.6, *p* = 0.028). The principal MRI and cognition associations were also generally preserved after controlling for GDS-15. In particular, hippocampal APT^#^ remained strongly associated with CDR-GS (r = 0.69, *p* < 0.001) and CDR-SB (r = 0.73, *p* < 0.001), and similar associations were observed for hippocampal NOE^#^ (r = 0.64 and 0.70, respectively; both FDR-adjusted *p* < 0.01). Hippocampal APT^#^ and NOE^#^ were also significantly associated with MMSE after adjustment for GDS-15 (both r = −0.45, *p* = 0.033). Complete results of the GDS-15-adjusted sensitivity analyses are provided in Supplementary Table S1.

### 3.4. Classification Performance of Single-Parameter and Multiparameter Models

**Figure 4:**
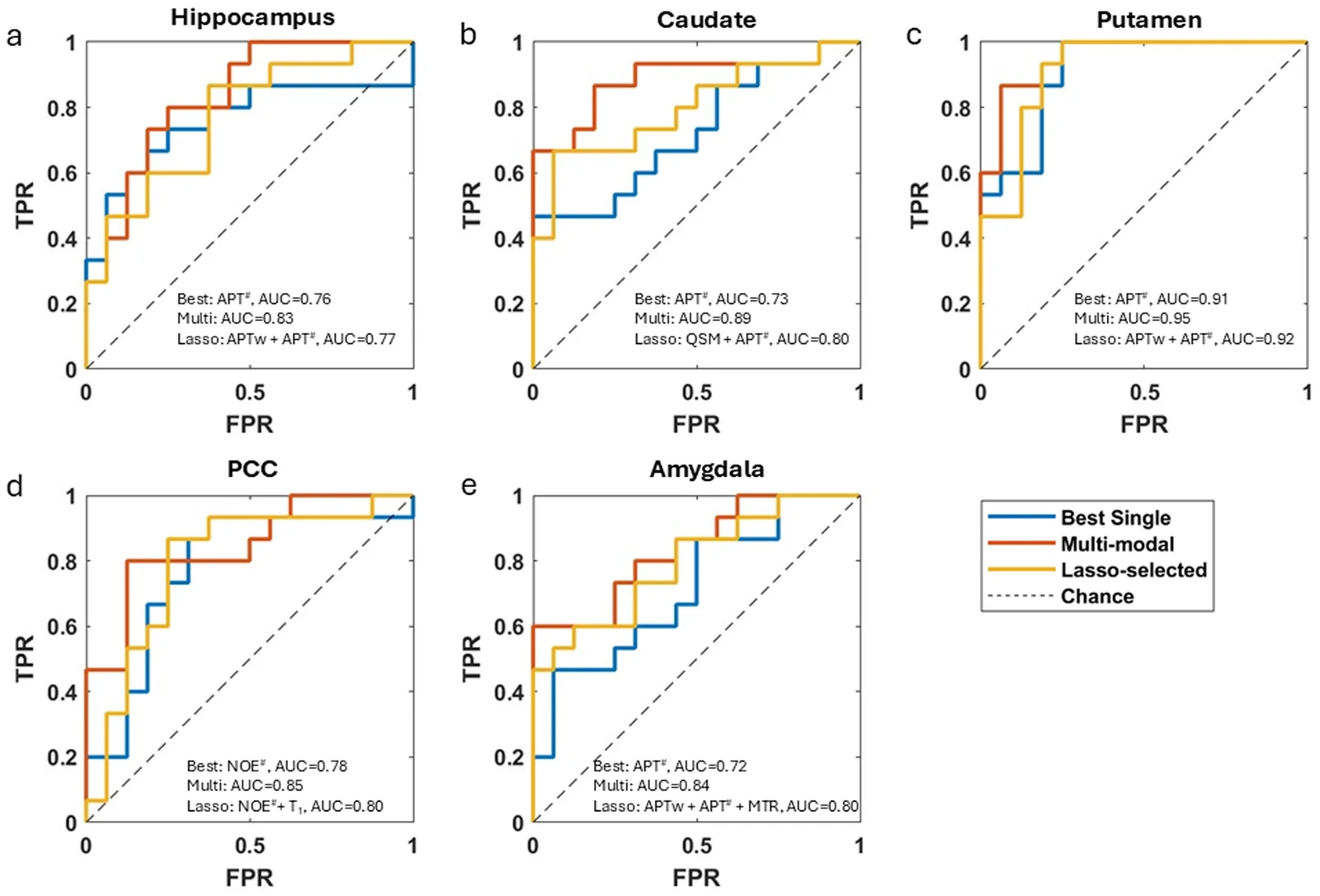
The ROC curves illustrate the comparative performance of single-parametric, multiparametric, and LASSO-selected models across regions of interest. (**a**) hippocampus; (**b**) caudate; (**c**) putamen; (**d**) PCC; and (**e**) amygdala.

Three predictive models were evaluated for each ROI: the single-parameter model (BestSingle), the full multiparameter model, and the LASSO-selected multivariate model. Table 4 summarizes their discriminative performance in distinguishing CN from MCI/mild dementia participants, with corresponding ROC curves shown in Figure 4. Across all ROIs, APT^#^ was the most frequently identified best-performing single parameter, yielding moderate-to-high classification accuracy with AUC values ranging from 0.72 to 0.92.

Across all ROIs, full multiparametric logistic regression models incorporating all available MRI measures generally demonstrated improved or comparable discriminative performance relative to single-parameter models. A statistically significant improvement of the full multiparameter model over the best single parameter was observed in the caudate (AUC: 0.89 vs. 0.73, *p* = 0.011), while differences in other ROIs did not reach statistical significance. These findings suggest that while APT-related measures provide strong individual discriminative power, multiparametric integration can further enhance classification performance, particularly in regions such as the caudate.

LASSO-based feature selection identified a reduced subset of informative parameters in most ROIs, typically retaining APT metrics with or without QSM. The classification performance of these LASSO-selected models was comparable to that of the best single-parameter models across ROIs, with no significant differences in pairwise AUC comparisons. In the amygdala, LASSO did not retain any predictors under cross-validation; therefore, LASSO-selected classification performance was not evaluated for this ROI.

## 4. Discussion

In this study, we systematically investigated group differences in advanced multiparametric MRI measures across multiple ROIs between CN and MCI/mild dementia participants, their relationships with cognitive impairment scores, as well as their utility for group discrimination between CN and MCI/mild dementia. Except for T_2_ and ADC, all MRI measures, including APTw, APT^#^ NOE^#^, MTR, T_1_, and QSM, demonstrated statistically significant group differences, with medium to very large effect sizes in at least one evaluated ROI. The associations between MRI biomarkers and clinical severity varied across MRI measures and brain regions. Among all ROIs, APT^#^ and NOE^#^ signals were consistently positively associated with CDR-GS and CDR-SB (significantly or insignificantly), MTR was consistently negatively associated with CDR (significantly or insignificantly), while APTw, T_1_, T_2_, QSM, and ADC showed relatively weaker or more complicated correlations with CDR. As expected, correlations with routine clinical screening MMSE were generally weaker and less consistent across ROIs than those with CDR-GS and CDR-SB. It was found that the TIV did not differ significantly between the two groups, indicating comparable overall brain size at the global level. Notably, among the evaluated MRI measures, APT^#^ demonstrated the most pronounced sensitivity to cognitive impairment across multiple regions. These findings suggest that APT imaging may be sensitive to molecular alterations associated with early cognitive decline, even when conventional structural or microstructural measures show less pronounced group differences. However, given the modest sample size and cross-sectional design, these observations should be considered preliminary and hypothesis-generating and require confirmation in larger independent cohorts.

Across most brain regions, APT^#^ and NOE^#^ signals showed the largest increases and positive correlations with CDR-GS and CDR-SB, whereas MTR exhibited an opposite direction and negative correlations. Theoretically, this indicates that increasing dementia severity comes with elevated mobile protein signals [39] and reduced macromolecular integrity [57,58]. These findings suggest a potential link between APT^#^ alterations and soluble Aβ and tau pathology during early AD [30]. Furthermore, PET studies have established that aggregated Aβ and tau are closely associated with disease progression and cognitive impairment in AD. As the disease progresses, the processes that lead to plaque formation also contribute to the ongoing presence and accumulation of toxic soluble oligomers, reinforcing the biological plausibility of linking CEST-based protein sensitivity to soluble Aβ and tau pathology [59]. Compared with molecular PET imaging, multiparametric MRI offers several practical advantages, including the absence of ionizing radiation, broad clinical availability, and the ability to characterize complementary tissue properties within a single imaging session. CEST imaging may provide sensitivity to changes in the tissue molecular environment that are not captured by conventional structural MRI. However, an important distinction is that the CEST-derived measures evaluated in the present study are not specific biomarkers of amyloid or tau pathology. In contrast, amyloid and tau PET directly target the defining protein aggregates of AD and therefore provide substantially greater molecular specificity [5,11]. Accordingly, the present MRI approach should be considered complementary to, rather than a replacement for, established molecular PET biomarkers. Future studies incorporating concurrent amyloid and tau PET will be necessary to determine the pathological correlates and added value of CEST-derived measures. Among all ROIs, increased hippocampal APT^#^ was most significant, reflecting early hippocampal vulnerability associated with AD. Increased hippocampal T_1_/T_2_ also showed positive correlations with CDR-GS and CDR-SB (significantly or insignificantly), potentially reflecting increased tissue water content or microstructural disorganization with disease progression [60]. Notably, these increased APT^#^ and T_1_/T_2_ observations in patients with early cognitive decline closely align with each other. Namely, increased tissue water content could imply an increase in mobile amide proton concentration, as reported in brain tumors and other diseases [47]. Although GDS-15 scores were modestly higher in the MCI/mild dementia group, depressive symptom burden was generally low, with only two participants scoring within the mild depressive symptom range. Furthermore, the principal associations between imaging and cognition, particularly those involving hippocampal APT^#^ and NOE^#^, were preserved after adjustment for GDS-15, suggesting that these relationships were not solely attributable to depressive symptoms.

Unexpectedly, PCC APTw was negatively correlated with CDR-GS (not significant) and CDR-SB (significant), suggesting reduced exchange-related signals with increasing dementia severity. The PCC is a key metabolic hub within the default mode network (DMN) and one of the earliest regions affected in AD, with structural and metabolic alterations associated with cognitive decline across the AD spectrum [61]. Functional and metabolic studies also show that hypometabolism and connectivity disruptions in the PCC correlate with lower MMSE and higher CDR, supporting the notion that reduced exchange-related multiparametric signals may reflect progressive dysfunction in this region [62]. Given the PCC’s central role in the DMN and its early involvement in AD, this finding may reflect diminished metabolic or protein-related activity in more advanced stages.

Previous studies have demonstrated increased QSM susceptibility in cognitively impaired individuals and its association with AD progression [63]. Hippocampal susceptibility has also been linked to cognitive decline independent of amyloid burden, supporting the potential detrimental effects of brain iron deposition other than amyloid pathology [64]. In the putamen, QSM showed significant positive correlations with CDR-GS and CDR-SB, and a significant negative correlation with MMSE, whereas T_2_ was positively correlated with MMSE. Given that the putamen is an iron-rich structure, the observed increase in QSM among cognitively impaired participants is consistent with previous studies reporting elevated susceptibility values in AD and their association with worse cognitive performance and greater disease severity [63,65,66]. In contrast, quantitative T_2_ studies have shown that longer T_2_ values are positively associated with MMSE scores in cognitively impaired individuals, suggesting relatively preserved tissue water content or microstructural integrity in those with better cognitive function [67].

However, it was unexpectedly found that hippocampal QSM was significantly positively correlated with MMSE scores. This counterintuitive finding contradicts a previous report [46] and should be interpreted with caution due to region-specific technical limitations. Structurally, the hippocampus is an elongated, slender formation with relatively low intrinsic magnetic susceptibility contrast compared to deep gray matter iron reservoirs like the caudate or putamen. During AD progression, progressive hippocampal atrophy may lead to the expansion of surrounding cerebrospinal fluid spaces, increasing the susceptibility of hippocampal measurements to PVE. Because our multiparametric analysis framework required all imaging measures to be co-registered and resampled onto the relatively thick CEST acquisition space, spatial downsampling may have introduced signal contamination from adjacent cerebrospinal fluid spaces and neighboring white matter tracts. Consequently, the observed positive association may not solely reflect pathological iron accumulation, but could also be influenced by a combination of diamagnetic protein deposition and partial volume effects associated with tissue atrophy. Therefore, this finding should be interpreted with caution.

Classification performance was evaluated using ROC-AUC analyses. Across most regions, the best single-parameter predictors were consistently APT^#^ and NOE^#^ (AUC = 0.72–0.92), highlighting their strong individual discriminative power. We previously reported a hippocampal APT^#^ AUC of 0.92 for group discrimination between CN and MCI/mild dementia [39], which was higher than 0.76 in this study. This discrepancy may be attributed to the different ROI selection: three coronal MRI slices used previously vs. the whole hippocampus based on all axial MRI slices in this study. Multiparametric integration provided modest improvements in certain regions, such as the caudate, but generally did not substantially outperform the best single-parameter predictors. LASSO-selected models yielded similar AUCs to the best single-parameter model and full multiparameter model, without statistically significant differences. Direct comparisons between the full multiparametric and LASSO-selected models were not performed, as the primary objective was to evaluate whether feature selection improved performance relative to the strongest single-parameter predictor. In some ROIs, LASSO feature selection retained no predictors, likely reflecting the limited sample size, multicollinearity among imaging features, or weak discriminative signal within those regions. Overall, these results suggest that APT^#^ and NOE^#^ provide robust classification ability, and that full multiparametric or LASSO-selected approaches offer only modest, region-dependent enhancements.

Unlike this study, several previous animal and human studies reported lower APT in the AD group, as discussed previously [39]. This discrepancy may be attributed to the different experimental settings (particularly higher B_0_ fields and lower B_1_ power), along with physiological differences (anesthesia, temperature, pH, and model-specific pathology) between human patients and mouse models. Furthermore, variations in disease stage and ROI definition should be considered. The amide proton pool in vivo is characterized by an exchange rate of tens to hundreds of Hz [68]. To ensure optimal radiofrequency label and transfer, a saturation power level of 1.5 to 2 µT is required. This range (1 µT ≈ 42.577 Hz) matches the amide proton exchange rate [69]. In this AD study, we used 1.5 µT for 1.5 s, slightly lower than the 2 µT/2 s recommended in brain tumor studies (where water T_1_ is longer). Notably, using these relatively higher saturation powers helps mitigate water T_1_ contamination in the resulting APT contrast [70]. Further investigations are needed to explain this contradiction.

Several limitations of this study should be acknowledged. First, the sample size was relatively modest, which may limit statistical power, precision of effect estimates, and the stability of multivariate classification models. Although several findings showed moderate-to-large effect sizes and consistent patterns across multiple brain regions, the corresponding estimates should be interpreted cautiously because small samples may yield unstable effect sizes and classification performance. We therefore consider the present findings exploratory and hypothesis-generating rather than confirmatory. Validation in larger, independent cohorts will be necessary to determine the robustness and generalizability of the observed imaging differences. Second, participants with MCI and mild dementia were combined into a single cognitively impaired group because of the limited sample size. Although this approach increased statistical feasibility, it also introduced clinical heterogeneity and may have obscured stage-specific imaging differences between MCI and mild dementia. The present findings therefore should not be interpreted as representing a single disease stage, and larger studies are needed to evaluate these clinical groups separately. Third, the cross-sectional design precludes direct inference about longitudinal disease progression, and future longitudinal studies are needed to determine whether APT^#^ and NOE^#^ changes precede cognitive decline within individuals. Fourth, molecular biomarkers of AD pathology, such as amyloid or tau PET or fluid biomarkers, were not available in the present cohort. Therefore, although the observed CEST alterations may reflect molecular and microenvironmental changes associated with cognitive decline, they cannot be attributed specifically to AD-related amyloid or tau pathology. Future studies incorporating molecular biomarker confirmation will be important to establish the pathological specificity of these MRI findings. Fifth, the APT sequence, with a thickness of 60 mm, consistently covered the entire hippocampus, rather than the whole brain. A whole brain APT sequence should be used in the future. The predefined ROI-based approach did not encompass all brain regions potentially relevant to cognitive impairment. In particular, the insula was not included in the present analysis, and future studies using broader regional or whole-brain analyses may help determine whether CEST and other quantitative MRI alterations extend to additional cortical regions [51]. Sixth, both elevated protein levels and pH can contribute to higher APT signals, and APT imaging of AD patients has a complicated contrast mechanism [23]. APT contrast is influenced by multiple biological factors, including mobile protein concentration, proton exchange rates, tissue pH, and other microenvironmental properties, which limits its pathological specificity. Although previous studies have reported only small pH fluctuations in AD (typically ranging from −0.07 to +0.01) [39], the present data do not allow us to determine the specific molecular source of the observed APT alterations. While soluble Aβ and tau may contribute to the mobile protein pool detectable by CEST-related mechanisms [33,34,35], the observed APT changes should not be interpreted as direct measures of either Aβ or tau pathology. Further studies combining CEST MRI with molecular biomarkers are needed to clarify the biological substrates underlying these signal alterations. Seventh, hippocampal QSM was unexpectedly found to be significantly positively correlated with MMSE scores, warranting a nuanced biophysical interpretation. Because our downstream multiparametric analysis required resampling the native 1-mm QSM data onto the 4-mm thick CEST slices, severe partial volume effects near the hippocampal boundaries were inevitable. This spatial downsampling likely introduced signal contamination from the adjacent white matter tracts and expanding cerebrospinal fluid spaces during disease progression, thereby confounding the true pathological correlation between hippocampal iron accumulation and cognitive decline. Future studies are needed to quantify the impact of partial volume effects and clarify the biological basis of hippocampal susceptibility changes in AD. Finally, the findings in this study were derived from a single-center cohort and scanner platform, and external validation in larger, multi-center datasets should be important to assess generalizability.

## 5. Conclusions

Our findings indicate that the relationships between MRI biomarkers and clinical scores vary by brain region and imaging parameter, reflecting the complex, non-linear nature of microstructural and molecular changes in early cognitive decline. Among the parameters examined, the APT^#^ signal showed the greatest sensitivity to cognitive function changes. This sensitivity likely indicates that consistent molecular alterations occur earlier than microstructural and macrostructural changes across multiple regions. Consequently, this suggests that APT^#^ is a promising imaging biomarker that is potentially reflecting soluble Aβ and tau species in brain areas particularly vulnerable to early pathological changes in AD.

## Figures and Tables

**Table 1:** Demographic and clinical characteristics of the CN (n = 18) and MCI/mild dementia (n = 18, including 14 MCI and 4 mild dementia) groups.

Variable	CN	MCI/Mild Dementia	*p*-Value
Age (mean ± SD; year)	70.3 ± 7.8	68.9 ± 8.8	0.606
Gender (Male/Female)	5/13	7/11	0.479
CDR-GS	0.00 ± 0.00	0.56 ± 0.16	**<0.001**
CDR-GS = 0	n = 18	n = 0	
CDR-GS ≥ 0.5	n = 0	n = 18	
CDR-SB	0.11 ± 0.21	2.11 ± 1.76	**<0.001**
CDR-SB = 0	n = 14	n = 0	
CDR-SB ≥ 0.5	n = 4	n = 18	
MMSE	28.8 ± 1.4	25.9 ± 3.5	**0.004**
GDS-15, median (IQR)	0.5 (0–2)	2 (1–3)	**0.032**
Major depressive disorder (Yes/No)	5/13	3/15	0.691
Anxiety disorder (Yes/No)	2/16	5/13	0.402
Hypertension (Yes/No)	7/11	10/8	0.505
Hypercholesterolemia (Yes/No)	7/11	12/6	0.181
Diabetes (Yes/No)	0/18	0/18	—
Vitamin B12 deficiency (Yes/No)	1/17	5/13	0.073
Thyroid disease (Yes/No)	3/15	1/17	0.289
TIV (mL)	1363.1 ± 130.9	1406.5 ± 142.3	0.332

Note: Significant differences (*p* < 0.05) are bolded.

**Table 2:** Cohen’s d effect size (FDR-corrected *p*-value) of CN vs. MCI/mild dementia for eight MRI measures across five ROIs.

MRI Measures	Hippocampus	Caudate	Putamen	PCC	Amygdala
APT^#^	**1.08 (0.007)**	**0.95 (0.013)**	**1.52 (<0.001)**	0.61 (0.091)	**0.94 (0.011)**
NOE^#^	**0.80 (0.047)**	**0.79 (0.031)**	0.41 (0.258)	**0.76 (0.037)**	0.19 (0.588)
APTw	0.48 (0.127)	0.19 (0.573)	0.59 (0.087)	−0.30 (0.372)	**0.75 (0.031)**
MTR	**−0.75 (0.049)**	−0.65 (0.059)	−0.58 (0.094)	−0.61 (0.076)	−0.64 (0.063)
T_1_	**0.82 (0.022)**	0.53 (0.119)	0.24 (0.477)	**0.79 (0.026)**	0.47 (0.177)
T_2_	0.67 (0.081)	0.25 (0.452)	0.26 (0.440)	0.23 (0.488)	0.41 (0.232)
QSM	−0.25 (0.548)	0.29 (0.393)	0.62 (0.072)	**−0.73 (0.039)**	−0.28 (0.410)
ADC	0.53 (0.126)	0.16 (0.645)	0.51 (0.150)	0.27 (0.438)	0.45 (0.205)

Note: Significant group differences (*p* < 0.05) are bolded.

**Table 3:** Pearson correlation coefficients (r) with corresponding *p*-values (FDR-corrected) between MRI measures across ROIs and cognitive scores (CDR and MMSE).

ROIs	MRI Measures	CDR-GS	CDR-SB	MMSE
Hipp.	APTw	r = 0.24, *p* = 0.183	r = 0.20, *p* = 0.288	r = −0.05, *p* = 0.768
	APT^#^	**r = 0.55, *p* = 0.008**	**r = 0.58, *p* = 0.003**	r = −0.19, *p* = 0.485
	NOE^#^	**r = 0.48, *p* = 0.021**	**r = 0.53, *p* = 0.006**	r = −0.15, *p* = 0.527
	MTR	**r = −0.39, *p* = 0.036**	**r = −0.41, *p* = 0.032**	r = 0.21, *p* = 0.485
	T_1_	**r = 0.41, *p* = 0.034**	r = 0.33, *p* = 0.080	r = −0.26, *p* = 0.485
	T_2_	**r = 0.38, *p* = 0.036**	**r = 0.40, *p* = 0.032**	r = −0.18, *p* = 0.485
	QSM	r = −0.19, *p* = 0.264	r = −0.19, *p* = 0.288	**r = 0.38, *p* = 0.047**
	ADC	r = 0.26, *p* = 0.180	r = 0.19, *p* = 0.288	r = −0.07, *p* = 0.768
Caudate	APTw	r = −0.01, *p* = 0.968	r = 0.05, *p* = 0.779	r = −0.07, *p* = 0.714
	APT^#^	**r = 0.42, *p* = 0.021**	**r = 0.38, *p* = 0.045**	r = −0.32, *p* = 0.246
	NOE^#^	**r = 0.43, *p* = 0.019**	**r = 0.37, *p* = 0.047**	r = −0.31, *p* = 0.246
	MTR	**r = −0.38, *p* = 0.049**	r = −0.34, *p* = 0.114	r = 0.28, *p* = 0.246
	T_1_	r = 0.27, *p* = 0.225	r = 0.21, *p* = 0.339	r = −0.26, *p* = 0.246
	T_2_	r = 0.12, *p* = 0.654	r = 0.12, *p* = 0.650	r = −0.11, *p* = 0.714
	QSM	r = 0.20, *p* = 0.375	r = 0.25, *p* = 0.270	r = −0.06, *p* = 0.714
	ADC	r = 0.06, *p* = 0.866	r = −0.06, *p* = 0.779	r = −0.09, *p* = 0.714
Putamen	APTw	r = 0.19, *p* = 0.357	r = 0.08, *p* = 0.627	r = −0.13, *p* = 0.504
	APT^#^	**r = 0.57, *p* = 0.004** * *	**r = 0.50, *p* = 0.022**	r = −0.28, *p* = 0.232
	NOE^#^	r = 0.27, *p* = 0.197	r = 0.31, *p* = 0.224	r = −0.06, *p* = 0.723
	MTR	r = −0.34, *p* = 0.117	r = −0.24, *p* = 0.267	r = 0.17, *p* = 0.428
	T_1_	r = 0.13, *p* = 0.519	r = 0.12, *p* = 0.627	r = 0.29, *p* = 0.232
	T_2_	r = 0.01, *p* = 0.956	r = −0.08, *p* = 0.627	**r = 0.39, *p* = 0.044**
	QSM	**r = 0.39, *p* = 0.048**	**r = 0.39, *p* = 0.046**	**r = −0.55, *p* = 0.001**
	ADC	r = 0.28, *p* = 0.197	r = 0.27, *p* = 0.253	r = 0.18, *p* = 0.428
PCC	APTw	r = −0.23, *p* = 0.238	**r = −0.35, *p* = 0.050**	r = 0.15, *p* = 0.602
	APT^#^	r = 0.26, *p* = 0.220	r = 0.10, *p* = 0.677	r = −0.14, *p* = 0.602
	NOE^#^	**r = 0.38, *p* = 0.041**	r = 0.27, *p* = 0.334	r = −0.19, *p* = 0.564
	MTR	r = −0.35, *p* = 0.103	r = −0.24, *p* = 0.334	r = 0.19, *p* = 0.564
	T_1_	r = 0.38, *p* = 0.103	r = 0.26, *p* = 0.334	r = −0.32, *p* = 0.490
	T_2_	r = 0.18, *p* = 0.327	r = 0.11, *p* = 0.677	r = −0.01, *p* = 0.986
	QSM	r = −0.28, *p* = 0.210	r = −0.14, *p* = 0.677	r = 0.19, *p* = 0.564
	ADC	r = 0.09, *p* = 0.604	r = 0.00, *p* = 0.998	r = 0.00, *p* = 0.986
Amygdala	APTw	**r = 0.38, *p* = 0.043**	r = 0.31, *p* = 0.183	r = −0.20, *p* = 0.287
	APT^#^	**r = 0.47, *p* = 0.044**	**r = 0.45, *p* = 0.047**	r = −0.26, *p* = 0.277
	NOE^#^	r = 0.13, *p* = 0.474	r = 0.17, *p* = 0.380	r = −0.07, *p* = 0.681
	MTR	**r = −0.40, *p* = 0.043**	**r = −0.39, *p* = 0.043**	r = 0.28, *p* = 0.277
	T_1_	r = 0.22, *p* = 0.381	r = 0.20, *p* = 0.380	r = −0.22, *p* = 0.277
	T_2_	r = 0.16, *p* = 0.417	r = 0.18, *p* = 0.380	r = −0.24, *p* = 0.277
	QSM	r = −0.16, *p* = 0.417	r = −0.24, *p* = 0.314	r = 0.29, *p* = 0.277
	ADC	r = 0.17, *p* = 0.417	r = 0.14, *p* = 0.422	r = −0.23, *p* = 0.277

Note: Significant associations (*p* < 0.05) are bolded.

**Table 4:** AUC (95% CI) and statistical comparisons for single-parametric, full multiparametric, and LASSO-selected models across ROIs.

ROIs	BestSingle	BestSingle AUC (95% CI)	Full-MP AUC (95% CI)	BestSingle vs. Full-MP *p*	LASSO-Selected	LASSO AUC	BestSingle vs. Lasso *p*
Hipp.	APT^#^	0.76 (0.55, 0.93)	0.83 (0.68, 0.96)	0.330	APT^#^; APTw	0.77	0.867
Caudate	APT^#^	0.73 (0.53, 0.89)	0.89 (0.74, 0.99)	**0.011**	APT^#^; QSM	0.80	0.085
Putamen	APT^#^	0.91 (0.79, 0.99)	0.95 (0.87, 1.00)	0.304	APT^#^; APTw	0.92	0.915
PCC	NOE^#^	0.78 (0.59, 0.94)	0.85 (0.70, 0.96)	0.380	NOE^#^; T_1_	0.80	0.558
Amygdala	APT^#^	0.72 (0.53, 0.88)	0.84 (0.68, 0.96)	0.095	APT^#^; APTw; MTR	0.80	0.286

Note: *p*-values (FDR-corrected) reflect comparisons of AUCs using the DeLong test. Significant difference (*p* < 0.05) is bolded. Abbreviations: BestSingle = the individual MRI parameter with the highest AUC within each ROI; Full-MP = models incorporating all available, full multiparametric MRI features; LASSO-selected = the subset of features retained by LASSO.

## Data Availability

The data that support the findings of this study are not publicly available due to patient privacy or ethical restrictions, but are available from the corresponding authors upon request.

## Supplementary Materials

The following supporting information can be downloaded at: https://www.mdpi.com/article/10.3390/neurolint18090177/s1, Table S1. GDS-15-adjusted partial correlations between MRI measures and cognitive scores across five ROIs.

## Abbreviations

The following abbreviations are used in this manuscript:

AD	Alzheimer’s disease
ADC	apparent diffusion coefficient
APT	amide proton transfer
APTw	amide proton transfer-weighted
AUC	area under the curve
CDR	clinical dementia rating
CDR-GS	CDR-global score
CDR-SB	CDR-sum of boxes
CEST	chemical exchange saturation transfer
CN	cognitively normal
DWI	diffusion-weighted imaging
FDR	false discovery rate
FLAIR	fluid-attenuated inversion recovery
GDS	geriatric depression scale
IQR	interquartile range
LASSO	least absolute shrinkage and selection operator
MCI	mild cognitive impairment
MMSE	mini-mental state examination
MTR	magnetization transfer ratio
NOE	nuclear Overhauser enhancement
PCC	posterior cingulate cortex
PET	positron emission tomography
QSM	quantitative susceptibility mapping
ROC	receiver operating characteristic
ROI	region of interest
T_1_w	T_1_-weighted
TIV	total intracranial volume
