# Supplementary material for: Assessing Microstructural and Molecular Brain Changes in Early Cognitive Decline Using Multiparametric 3T Magnetic Resonance Imaging
Source: Neurol Int. 2026 Sep 18;18(9):177. doi: 10.3390/neurolint18090177 (PMC13610878; doi:10.3390/neurolint18090177)
Supplement: Supplementary file 1 [file neurolint-18-00177-s001.zip › neurolint-4531843-supplementary.pdf]

Table S1. GDS-15-adjusted partial correlations between MRI measures and cognitive scores across five ROIs.

ROIs	MRI measures	CDR-GS	CDR-SB	MMSE
Hippocampus	APTw	r = 0.27, p = 0.121	r = 0.23, p = 0.205	r = −0.10, p = 0.581
	APT#	**r = 0.69, p < 0.001**	**r = 0.73, p < 0.001**	**r = −0.45, p = 0.033**
	NOE#	**r = 0.64, p < 0.001**	**r = 0.70, p < 0.001**	**r = −0.45, p = 0.033**
	MTR	**r = −0.53, p = 0.002**	**r = −0.53, p = 0.002**	**r = 0.41, p = 0.033**
	T_1	**r = 0.44, p = 0.010**	r = 0.35, p = 0.062	r = −0.30, p = 0.101
	T_2	**r = 0.53, p = 0.002**	**r = 0.53, p = 0.002**	**r = −0.40, p = 0.033**
	QSM	r = −0.12, p = 0.492	r = −0.12, p = 0.481	r = 0.30, p = 0.101
	ADC	r = 0.27, p = 0.121	r = 0.23, p = 0.205	r = −0.10, p = 0.581
Caudate	APTw	r = −0.05, p = 0.785	r = 0.02, p = 0.925	r = −0.01, p = 0.943
	APT#	**r = 0.45, p = 0.023**	**r = 0.41, p = 0.048**	**r = −0.41, p = 0.049**
	NOE#	**r = 0.47, p = 0.022**	**r = 0.42, p = 0.048**	**r = −0.44, p = 0.046**
	MTR	**r = −0.46, p = 0.022**	**r = −0.41, p = 0.048**	**r = 0.43, p = 0.046**
	T_1	r = 0.29, p = 0.173	r = 0.23, p = 0.292	r = −0.31, p = 0.131
	T_2	r = 0.15, p = 0.501	r = 0.15, p = 0.519	r = −0.17, p = 0.510
	QSM	r = 0.18, p = 0.468	r = 0.24, p = 0.292	r = −0.01, p = 0.943
	ADC	r = 0.09, p = 0.685	r = −0.04, p = 0.925	r = −0.15, p = 0.550
Putamen	APTw	r = 0.14, p = 0.492	r = 0.03, p = 0.864	r = −0.04, p = 0.823
	APT#	**r = 0.54, p = 0.009**	**r = 0.47, p = 0.045**	r = −0.19, p = 0.473
	NOE#	r = 0.29, p = 0.155	r = 0.32, p = 0.173	r = −0.08, p = 0.733
	MTR	r = −0.39, p = 0.078	r = −0.27, p = 0.173	r = 0.25, p = 0.385
	T_1	r = 0.27, p = 0.155	r = 0.24, p = 0.206	r = 0.14, p = 0.545
	T_2	r = 0.04, p = 0.808	r = −0.06, p = 0.832	r = 0.38, p = 0.090
	QSM	**r = 0.43, p = 0.045**	r = 0.31, p = 0.173	r = −0.41, p = 0.090
	ADC	r = 0.32, p = 0.152	r = 0.30, p = 0.173	r = 0.19, p = 0.473
PCC	APTw	r = −0.20, p = 0.268	**r = −0.38, p = 0.050**	r = 0.11, p = 0.652
	APT#	r = 0.26, p = 0.225	r = 0.09, p = 0.714	r = −0.14, p = 0.652
	NOE#	**r = 0.37, p = 0.046**	r = 0.25, p = 0.353	r = −0.16, p = 0.652
	MTR	r = −0.36, p = 0.096	r = −0.24, p = 0.353	r = 0.21, p = 0.652
	T_1	r = 0.36, p = 0.096	r = 0.23, p = 0.353	r = −0.29, p = 0.652
	T_2	r = 0.24, p = 0.225	r = 0.16, p = 0.568	r = −0.10, p = 0.652
	QSM	r = −0.23, p = 0.225	r = −0.09, p = 0.714	r = 0.13, p = 0.652
	ADC	r = 0.13, p = 0.448	r = 0.03, p = 0.871	r = −0.04, p = 0.821
Amygdala	APTw	**r = 0.43, p = 0.044**	r = 0.26, p = 0.319	r = −0.10, p = 0.569
	APT#	**r = 0.48, p = 0.019**	**r = 0.46, p = 0.029**	r = −0.28, p = 0.298
	NOE#	r = 0.19, p = 0.447	r = 0.24, p = 0.319	r = −0.23, p = 0.312
	MTR	**r = −0.47, p = 0.019**	**r = −0.46, p = 0.029**	r = 0.41, p = 0.096
	T_1	r = 0.22, p = 0.385	r = 0.19, p = 0.319	r = −0.23, p = 0.312
	T_2	r = 0.16, p = 0.455	r = 0.19, p = 0.319	r = −0.28, p = 0.298
	QSM	r = −0.10, p = 0.582	r = −0.19, p = 0.319	r = 0.20, p = 0.312
	ADC	r = 0.12, p = 0.582	r = 0.09, p = 0.605	r = −0.17, p = 0.378

Note: Values are partial Pearson correlation coefficients (r) with corresponding Benjamini–Hochberg false discovery rate (FDR)-adjusted p-values, controlling for GDS-15 score. FDR correction was applied across the eight MRI measures within each ROI and cognitive outcome. Analyses were performed using complete cases; therefore, sample sizes varied across MRI measures because of missing imaging or GDS-15 data. Significant associations after FDR correction ($p < 0.05$) are bolded.
